# Increased stimulatory capacity of antigen presenting cells at the site of autoimmune inflammation interferes with regulatory T cell function

**DOI:** 10.1186/1546-0096-9-S1-P280

**Published:** 2011-09-14

**Authors:** Femke Van Wijk, E Wehrens, C Duurland, B Vastert, M Klein, J Meerding, W De Jager, B Prakken

**Affiliations:** 1 University Medical Centre Utrecht, UTRECHT, The Netherlands

## Background

FOXP3+ regulatory T cells (Treg) are critical in maintaining self tolerance and are therefore considered important targets for the treatment of autoimmune disease. However, environmental factors at the site of autoimmune inflammation, such as enhanced costimulatory potential of antigen presenting cells (APC) and increased proinflammatory cytokine production, can negatively affect Treg function, thereby limiting effectiveness of these Treg targeted approaches.

## Aim

Here we studied the phenotype of APC present at the site of inflammation in patients with Juvenile Idiopathic Arthritis (JIA) and investigated whether these cells can interfere with Treg mediated suppression.

## Methods

Mononuclear cells were isolated from peripheral blood (PB) of healthy controls (HC) and from paired PB and synovial fluid (SF) of JIA patients. The phenotype of APC was analysed using flow cytometry. In vitro suppression assays were performed to study T cell activation and Treg mediated suppression in the presence of SF and PB derived APC.

## Results

Monocytes from the site of inflammation displayed a more pro-inflammatory phenotype, with significantly increased costimulatory molecule expression, compared to monocytes from PB. In line with this pro-inflammatory phenotype, SF APC induced enhanced proliferation of effector cells and decreased suppression of effector cell proliferation in the presence of Treg.

## Conclusions

APC from the site of inflammation have an enhanced stimulatory capacity that interferes with Treg mediated suppression. Therefore, this increased stimulatory potential should be targeted as well, in order for a Treg enhancing approach to be fully effective in the treatment of autoimmune inflammation.

